# Sustainability of soil organic carbon in consolidated gully land in China’s Loess Plateau

**DOI:** 10.1038/s41598-020-73910-7

**Published:** 2020-10-09

**Authors:** Qina Yan, Praveen Kumar, Yunqiang Wang, Yali Zhao, Henry Lin, Qihua Ran, Zhisheng An, Weijian Zhou

**Affiliations:** 1 Department of Civil and Environmental Engineering, University of Illinois at Urbana-Champaign, Urbana, IL USA; 2 Department of Atmospheric Sciences, University of Illinois at Urbana-Champaign, Urbana, IL USA; 3 State Key Laboratory of Loess and Quaternary Geology, Institute of Earth Environment, Chinese Academy of Sciences, Xi’an, Shaanxi China; 4 CAS Center for Excellence in Quaternary Science and Global Change, Chinese Academy of Sciences, Xi’an, China; 5 Department of Ecosystem Science and Management, Pennsylvania State University, University Park, PA USA; 6 Institute of Hydrology and Water Resources, College of Civil Engineering and Architecture, Zhejiang University, Hangzhou, China; 7 Present Address: Lawrence Berkeley National Laboratory, Berkeley CA, USA

**Keywords:** Carbon cycle, Hydrology, Geomorphology

## Abstract

Massive gully land consolidation projects, launched in China’s Loess Plateau, aim to restore 2667 $\mathrm{km}^2$ agricultural lands in total by consolidating 2026 highly eroded gullies. This effort represents a social engineering project where the economic development and livelihood of the farming families are closely tied to the ability of these emergent landscapes to provide agricultural services. Whether these ‘time zero’ landscapes have the resilience to provide a sustainable soil condition such as soil organic carbon (SOC) content remains unknown. By studying two watersheds, one of which is a control site, we show that the consolidated gully serves as an enhanced carbon sink, where the magnitude of SOC increase rate (1.0 $\mathrm{g\,C}/\mathrm{m}^2/\mathrm{year}$) is about twice that of the SOC decrease rate (− 0.5 $\mathrm{g\,C}/\mathrm{m}^2/\mathrm{year}$) in the surrounding natural watershed. Over a 50-year co-evolution of landscape and SOC turnover, we find that the dominant mechanisms that determine the carbon cycling are different between the consolidated gully and natural watersheds. In natural watersheds, the flux of SOC transformation is mainly driven by the flux of SOC transport; but in the consolidated gully, the transport has little impact on the transformation. Furthermore, we find that extending the surface carbon residence time has the potential to efficiently enhance carbon sequestration from the atmosphere with a rate as high as 8 $\mathrm{g\,C}/\mathrm{m}^2/\mathrm{year}$ compared to the current 0.4 $\mathrm{g\,C}/\mathrm{m}^2/\mathrm{year}$. The success for the completion of all gully consolidation would lead to as high as 26.67 $\mathrm{Gg\,C}/\mathrm{year}$ sequestrated into soils. This work, therefore, not only provides an assessment and guidance of the long-term sustainability of the ‘time zero’ landscapes but also a solution for sequestration $\hbox {CO}_2$ into soils.

## Introduction

**Figure 1:**
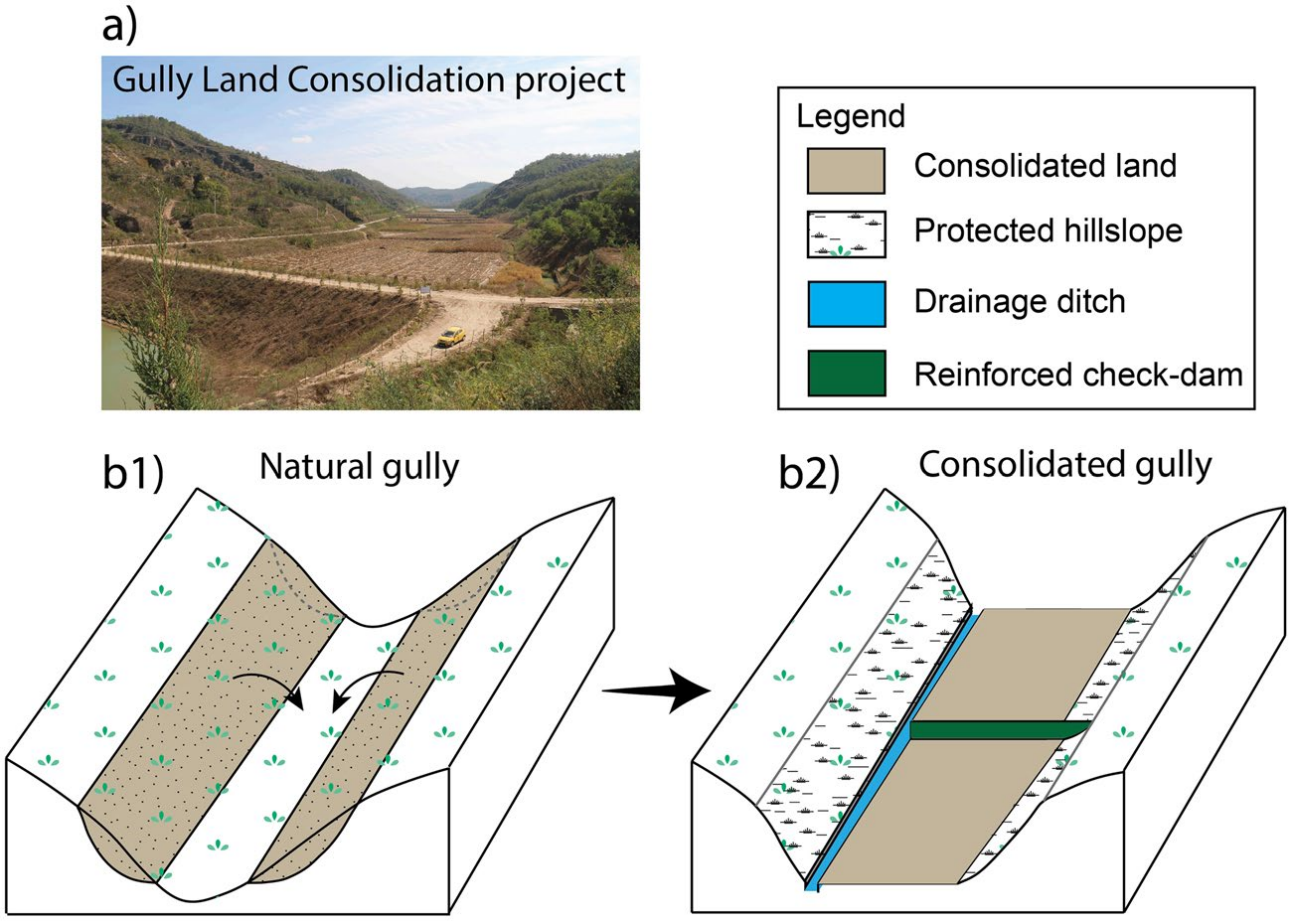
Illustration of the gully land consolidation project in China’s Loess Plateau. The major constructions include: excavating soils from the hillslope and compacting them inside the gully, protecting the excavated hillslope from erosion by growing plants and building walls, building drainage ditches, and reinforcing existing or developing new check-dams. (**a**) A field view of a consolidated gully land in a main stem gully area (photo credit: Praveen Kumar). (**b1**) Schematic of topography before the consolidation. The highlighted area with brown color represents the soil excavated from the hillslope. (**b2**) Schematic of the topography after compacting grounds in the consolidated gully.

In China’s Loess Plateau, human activities have frequently altered the landscapes over thousands of years^1–3^. One of the recently initiated projects, gully land consolidation (GLC) (Fig. 1a), aims to restore substantial farmlands within significantly eroded large gullies—first creating 377.8 $\mathrm{km}^2$ in Yan’an City^4,5^ and then up to 2667 $\mathrm{km}^2$ among the whole loess plateau^6^. This project was launched in 2011 to complement the Grain-for-Green (GFG) project^5^. The GFG project traded 2.69 $\times$ 10$^6$ $\mathrm{km}^2$ farmlands, comprising of 75% hilly farmlands and 46% Gobi-arid farmlands, for grasslands, shrubs, and forests to rehabilitate the ecosystem since 1999^7^. Both satellite data and local measurement show that the GFG project resulted in a significant increase of vegetation cover, reduced surface runoff, and more importantly, reduced soil erosion rate consistent with historic low values^8–13^, over the past three decades. However, because the GFG project covers 25 provinces and directly affects 124 million farmers^7^, the shortage of farmlands as well as the increasing population results in deficits of food supply and livelihood for both local and surrounding regions^8^. As a result, the GLC project emerged to ameliorate these negative outcomes. It aims to integrate the interests of the government to sustain the accomplishment from the GFG project and the community of farmers to secure food supply and increase income. Moreover, expected significant social benefits include supporting modern industrial agriculture, boosting the resilience of rural communities, and balancing resources between urban and rural areas^5^. The success of the GLC project relies on whether these massively engineered landscapes are capable of providing targeted hydrological, biogeochemical, and geomorphological functionality at present and in the future. Soil sustainability, therefore, plays a central role in these critical zones^14^. In this study, we use soil organic carbon (SOC) as an indicator of soil health and fertility^15–17^ and conduct numerical modeling to assess how SOC dynamics respond in the consolidated gully and surrounding watershed in both the short-term and long-run.

Consolidation of fragmented land parcels for agriculture has a long history worldwide in plain regions, but the GLC project instead targets the highly eroded gullies in a hilly plateau. In China’s Loess Plateau, highly erodible loessial sediment are very sensitive to erosion from surface flow, which has formed extensive large scale gullies, particularly in the hilly plateau region. Among these gullies^2^, 270,000 of them are longer than 500 m. These ravine-like landscapes hinder vegetation restoration and agricultural development. One approach for managing soil loss has been to build check-dams inside the gully to trap sediment and prevent the gully from further expansion^2^—a strategy that dates back over 400 years. This approach provides potential farmable lands because the local relief is reformed to be relatively flat, and the trapped soil particles from upstream have relatively small sizes that favor SOC conservation^3^. Further, revegetation under the GFG project has reduced the erosion rate inside gullies along with an increased net primary production and declined surface runoff^13, 18^, which creates favorable conditions for creating farmland inside the historically eroded gully.

The GLC project started in Yan’an city^5^ in 2012. After a comprehensive assessment of the local land conditions, 2026 gullies were identified as ideal locations. The gully consolidation process includes four construction steps: (1) excavating soils from hillslopes and compacting them in the gully area; (2) protecting the hillslopes from erosion by growing plants and building walls as needed; (3) creating drainage ditches; and (4) building new check-dams or reinforcing existing ones (Fig. 1). Depending on the pre-construction condition and size of the gully, steps 2 and 3 may not be necessary for all of the consolidated gullies. However, even with careful site selection and engineering design, the sustainability of these landscapes to perform the intended functions needs to be assessed, and some crucial questions remain unanswered. For example, what is the difference between consolidated gully watershed and natural watershed in terms of the soil organic carbon redistribution and turnover for both a long-term evolution and an intra-annual cycle? What is the impact of soil materials transported from upland on the consolidated gully area? How is the effectiveness of litter input and residence time management on maintaining soil fertility with regards to organic carbon?

**Figure 2:**
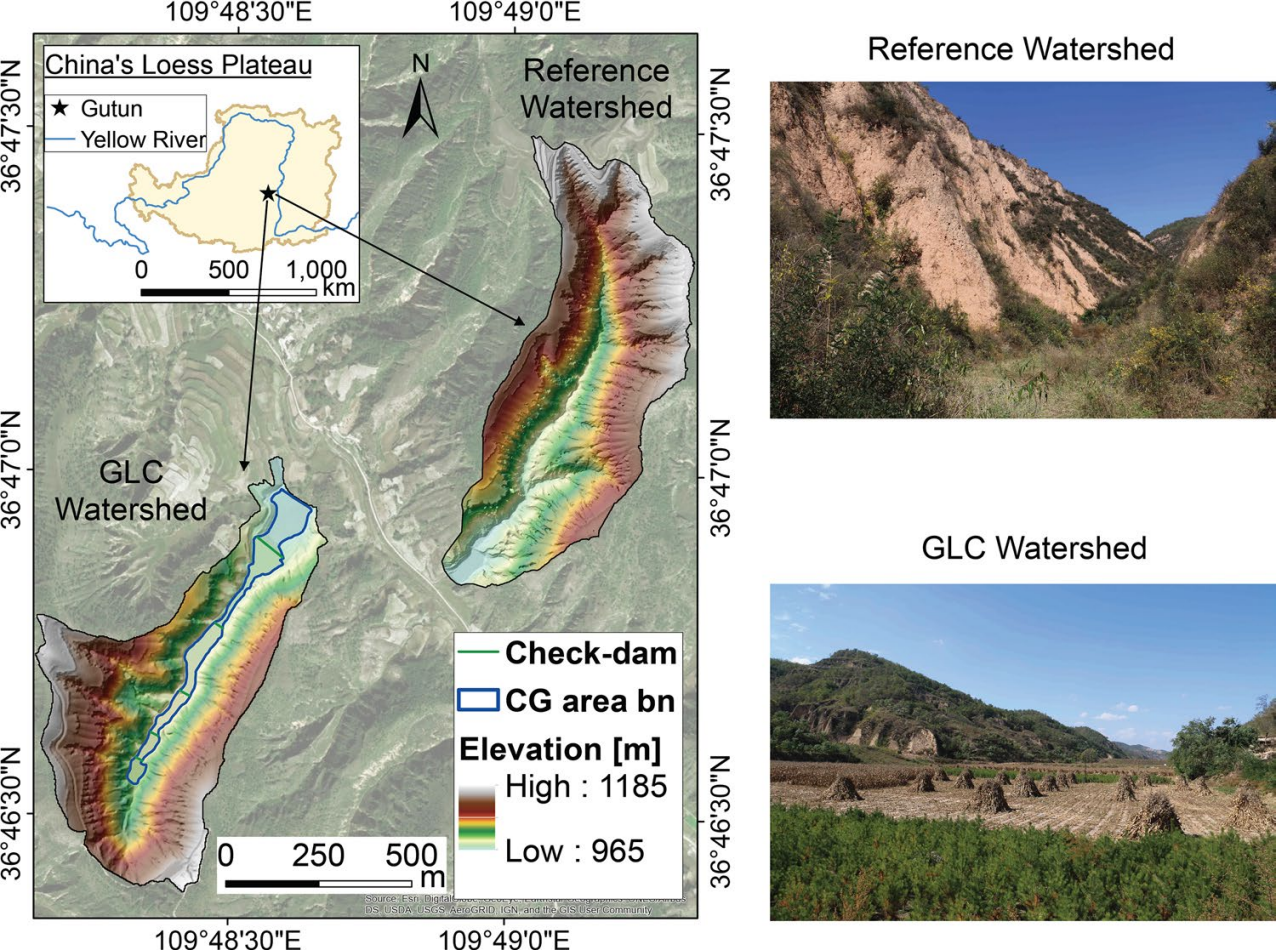
Map of the two study watersheds at Gutun station in China’s Loess Plateau. Left: 2-m digital elevation model (DEM). Created using ArcGIS version 10.5. The western watershed is subjected to the gully land consolidation project, henceforth named the GLC Watershed. The consolidated gully within this watershed is highlighted with a blue boundary. In this consolidated gully, five check-dams are built which divide the entire gully into six relatively flat parcels of farmlands. The eastern watershed is under natural conditions. Right: field views of the two watersheds. Photos taken in October 2017, photo credit: Praveen Kumar.

To address these questions, we apply our simulations in two watersheds in Gutun station (Fig. 2). The one to the west corresponds to the GLC project (constructed in 2013 and subjected to land consolidation and check dam construction), hereafter referred to as the GLC Watershed. The one to the east is a natural watershed, hereafter referred to as the Reference Watershed. Because these two watersheds nearby (420 km away) have similar size ( 0.45 km$^2$ and  0.43 km$^2$ for GLC and Reference Watershed, respectively), landcover (Figure S7b), and soil textures (31% sand, 4% clay, and 65% silt; 26% sand, 5% clay, and 69% silt for GLC and Reference Watershed, respectively), we assume that they have undergone similar erosion and depositional history before the GLC project took place^19^. We assume that the SOC content below the Bt horizon is relatively stable compared to the surface fluctuation^20^, so we only consider the SOC stock change above the Bt horizon as the total SOC stock change in a whole soil column. Hence, in our simulation results, we provide the SOC stock change ($\mathrm{kg\,C}/\mathrm{time}$) not the total SOC stock (kg C). Also, the weathering rate of clay in 50 years would have little impact on SOC content because clay formation would need a much longer time horizon^21–23^.

Here, we use a recently developed and validated process-based quasi-3D model, SCALE (Soil Carbon and Landscapes co-Evolution^24^). This model simulates five key processes: soil transport and associated landscape evolution, SOC lateral transport, overland flow, soil moisture dynamics, and SOC biogeochemical transformation. This model is designed to capture the co-evolution of landscape (and associated SOC surface removal and burial) and below-surface SOC decomposition throughout the vertical soil column, which simulates dynamic SOC profiles across a watershed. Simulations are performed using a discretization with 2 $\times$ 2 $\mathrm{m}^2$ grid, based on the finest resolution elevation data available, and at daily time scales. The inputs for initial conditions and forcing data can be found in the Methods section and Supplementary Information. Our simulation results assess the sustainability of these landscapes through the SOC dynamics over the short-term (intra-annual) and long-run (i.e., 50-years) through a comparative analysis of the GLC Watershed excluding the consolidated gully, the consolidated gully, and the Reference Watershed. Moreover, we elucidate the possible outcomes of SOC stock change by simulating different land management practices.

## SOC stock change after a 50-years evolution

**Figure 3:**
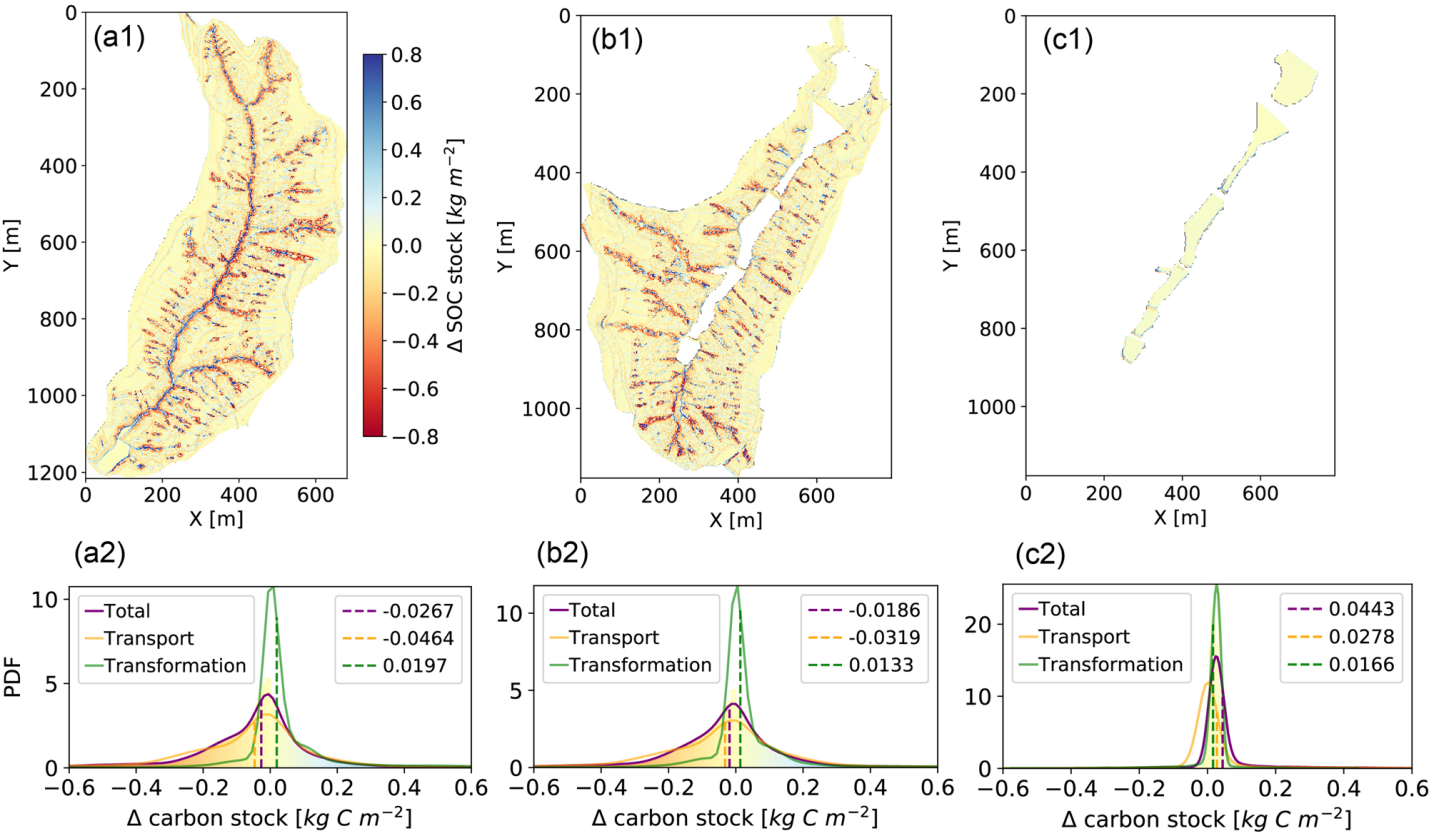
The total SOC stock change after a 50-years evolution in the Reference Watershed (**a**), GLC Watershed excluding the consolidated gully (**b**), and the consolidated gully only (**c**). (**a1**–**c1**) Spatial map of total SOC stock change. (**a2**–**c2**) Probability distribution functions (PDFs). The dash lines represent spatial mean values. The purple line corresponds to the PDF of total SOC stock change, the orange line corresponds to lateral transport, and the green line corresponds to biogeochemical transformation.

SOC stock at each 2-D surface grid box is obtained by integrating the SOC concentration throughout a soil column. The range of SOC stock changes in the Reference and GLC watersheds (without gully lands), projected for a 50-years co-evolution of landscape and biogeochemical transformation, is within − 0.8 to 0.8 $\mathrm{kg\,C}/\mathrm{m}^2$ (Fig. 3a1–b1). This range is within the same order of magnitude compared to those derived from a national survey and simulations^25,26^. The spatial mean carbon stock losing rate in the GLC Watershed (i.e., − 0.0186 $\mathrm{kg\,C}/\mathrm{m}^2$) is smaller than the Reference Watershed (i.e., − 0.0267 $\mathrm{kg\,C}/\mathrm{m}^2$) due to the construction of the consolidated gully land in that the erosion rate slows down near the boundaries of the main valley^27^. The soil thickness change due to surface erosion and deposition during the same 50-years period is within − 0.4 to 0.4 m (see more information in Figure S1 in the Supplementary Information), which is also consistent with other studies^26,28^. The SOC stock change at each 2$\times$2 $\mathrm{m}^2$ grid box is the net of the surface SOC transport and SOC transformation (see more in the Supplementary Information, Figure S2a). SOC transport is physically redistributed due to the movement of soil through detachment from one place (erosion), transport, and burial elsewhere (deposition), therefore, referred to as the SOC lateral flux. SOC biogeochemical transformation is a soil-atmosphere carbon exchange by which the soil column indirectly accumulates $\hbox {CO}_2$ via inputs from plant residues and directly releases $\hbox {CO}_2$ back to the atmosphere through microbial decomposition^29–31^, therefore, referred as the SOC vertical flux. Many studies have revealed the significant interactions among the two processes, particularly in fast erosional areas^24,25,30, 32–34^. We disentangle the SOC transport and transformation fluxes at each 2-D grid point and show the spatial statistics of transport, transformation, and total (their sum) in Fig. 3a2–c2.

Comparing the spatial means among all of the three areas, we find that the magnitude of SOC transport flux is about twice that of transformation (Fig. 3a2–c2), which indicates that the transport plays a stronger role for SOC stock change after a 50-years evolution. In the natural watersheds, the spatial mean of SOC transport is negative, meaning that the watershed as a whole is undergoing erosion; but the spatial mean of SOC transformation is positive, meaning that the watershed as a whole is a net carbon sink for the atmospheric $\hbox {CO}_2$. In the consolidated gully, however, both the transport and transformation result in positive SOC stock change, which suggests the consolidated gully is not only a SOC depositional area but also a carbon sink for the atmospheric $\hbox {CO}_2$ (Fig. 3c2). Furthermore, in the natural watersheds, the high spatial variability of SOC transport flux (Fig. 3a2–c2) implies that many of the highly eroded soils, which carry organic carbon, detached from erosional sites are deposited at a nearby location within the watersheds but not carried away out of the system^30^.

**Figure 4:**
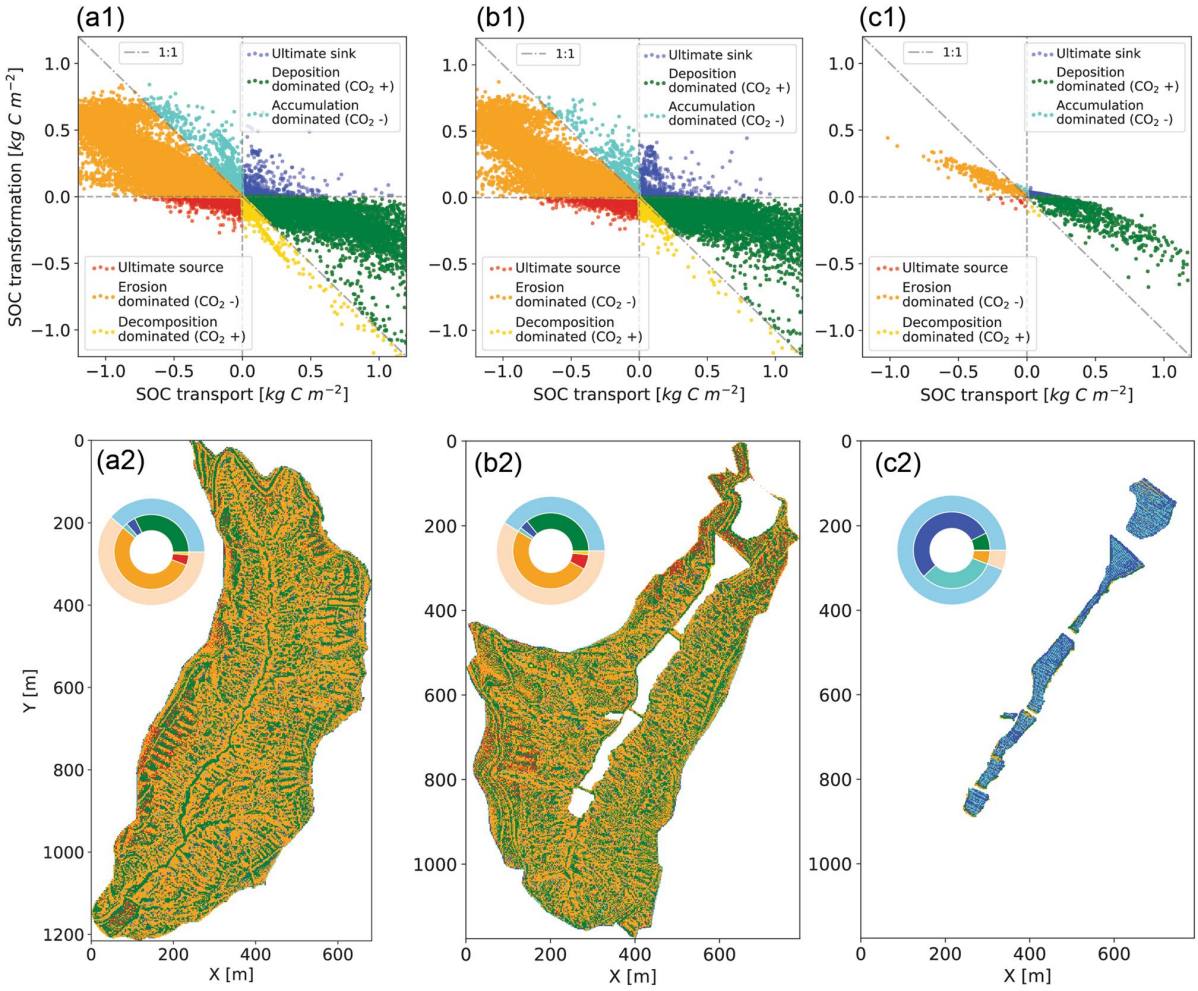
Comparison of SOC flux from lateral transport and biogeochemical transformation after a 50-years co-evolution. (**a1**–**c1**) The *x*-axis represents lateral SOC transport, where a positive value indicates net deposition and a negative value indicates net erosion. The *y*-axis represents the vertical soil-atmosphere carbon exchange through biogeochemical transformation, where a positive exchange indicates a net carbon flux to soils (sink), and a negative value indicates a net carbon flux to the atmosphere (source). $x+y >0$ corresponds to SOC gain, which is the three groups in cool colors above the $y = -x$ line; $x+y<0$ corresponds to SOC loss, which are the three groups in warm colors below the $y=-x$ line. If $x>0$ and $y>0$, the sites are called ultimate sink (blue); if $x<0$ and $y<0$, the sites are called ultimate source (red). If $|x| > |y|$, transport (erosion and deposition) is dominant, otherwise transformation (decomposition and accumulation) is dominant. If a site is a carbon source to the atmosphere, then it is labeled as ‘$\hbox {CO}_2$+’; otherwise, a sink is labeled as ‘$\hbox {CO}_2$−’. (**a2**–**c2**) The spatial map of the six groups distributed in the watersheds. The pie charts represent the percent area of each zone. The outside light blue band represents the percentage area of the SOC sink ($x+y>0$), and the light orange band represents the percentage area of SOC source ($x+y<0$). Colors in the inner band correspond to those in the top row.

The flux of SOC transformation is strongly controlled by the flux of transport in natural watersheds but not necessarily in the consolidated gully, even though the magnitude of transport flux is stronger in the consolidated gully. The general trend of the ratio of transformation to transport for all grid cells is less than 1 (Fig. 4a1–c1). We investigate in more detail by dividing the whole region into two large groups—carbon gain sites (cool colors); and carbon loss sites (warm colors). Based on the signs and magnitudes of the two fluxes, each group is further divided into three subgroups: (1) ultimate sink (blue), where transport and transformation both result in SOC gain, or ultimate source (red), where the two fluxes both result in SOC loss; (2) deposition (green) or erosion dominated (orange), where the signs of the two fluxes are opposite, but transport flux has a higher magnitude than transformation; and (3) accumulation (light blue) or decomposition (yellow) dominated, where the signs of the two fluxes are opposite, but transformation flux has higher magnitude than transport (more information can be found in Supplementary Information, Figure S2). The percentage area of the six categories is shown in Fig. 4a2–c2. In natural watersheds, slightly over half of the total areas correspond to SOC stock loss. Among these carbon loss sites, the majority of them are erosion dominated, which also serve as net carbon sinks for the atmospheric $\hbox {CO}_2$. Among carbon gain sites, the majority of them are deposition dominated sites, which also serve as net carbon sources to the atmospheric $\hbox {CO}_2$. In consolidated gullies, the majority of the area is gaining carbon. Interestingly, the top two largest groups (approximately 90%) are either ultimate sink or accumulation dominated sites. This significant difference compared to natural watersheds is because the majority of the areas have sufficiently small fluxes associated with erosion and deposition. The SOC transformation becomes the dominant process and has little influence from transport, which can be future explained in the intra-annual SOC dynamics.

## Intra-annual dynamics of soil organic carbon

**Figure 5:**
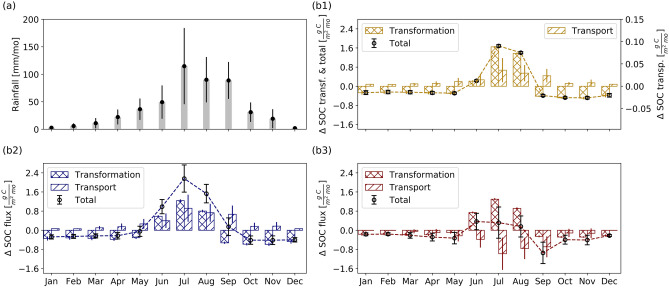
Intra-annual variation of precipitation and monthly mean SOC stock change of the consolidated gully and the Reference Watershed for a 50-years co-evolution of landscape and biogeochemical transformation. An error bar corresponds to the standard deviation of values for the same month. (**a**) Monthly mean precipitation. (**b1**–**b3**) Monthly mean SOC fluxes of transformation, transport, and total for: (**b1**) the consolidated gully (note, the transport flux follows the *y*-axis on the right); (**b2**) depositional zone; and (**b3**) erosional zone (more information about the two zones can be found in Figure S4 in Supplementary Information).

The intra-annual dynamics of SOC are mainly driven by the biogeochemical transformation in the consolidated gully, but are driven by both transformation and transport processes in natural watersheds (Fig. 5). The summer monsoon season^35^ drives the pattern of rainfall variability (Fig. 5a), which directly determines the pattern for SOC transport flux (Fig. 5b1–b3) in that the annual cycle and the temporal variation for each month (error bars) are consistent with that of rainfall. On the other hand, the temporal variability in SOC transformation across different months is more constant with smaller error bars because of the same quality of litter input among each annual cycle (Figure S3 in Supplementary Information). In the consolidated gully (Fig. 5b1), the flux of SOC transformation is one order of magnitude higher than the transport (or net deposition in this case). Therefore, the intra-annual cycle of SOC dynamics is mainly driven by the biogeochemical transformation, which explains why the SOC transport has little influence on transformation fluxes over long-term (50-years) evolution.

In contrast to the consolidated gully, the intra-annual dynamics of SOC in watersheds show evidence of the influence of both biogeochemical transformation and intense SOC transport. We use the Reference watershed to represent the dynamics of natural watersheds because Reference and GLC watersheds have very similar evolutionary processes in terms of the fate, rate, and magnitude of SOC dynamics (which can be seen from the similar shapes of PDFs in Fig. 3a2,b2 and Figure S1a2,b2). We choose two representative zones from the PDF of soil depth change: depositional zone (Fig. 5b2) and erosional zone (Fig. 5b3) (see details in Figure S6 in Supplementary Information). During the growing season, SOC transformation fluxes among erosional and depositional zones are similar, but during the non-growing period, SOC transformation flux (or the release of $\hbox {CO}_2$ by decomposition) is larger in the depositional zone. This is because the surface redistribution of SOC at the depositional zone provides more available SOC for decomposition, whereas in the erosional zone, the net erosion results in a favorable soil condition for SOC accumulation through biogeochemical transformation^30, 36^.

## Land management practices for sustainability in the consolidated gully

Since the SOC transformation in the consolidated gully has little influence from SOC transport during both the intra-annual cycle and over the long-run, we test different land management practices in the consolidated gully, intending to explore options for maintaining or even increasing the SOC stock over the long-run by managing soil carbon. Land management practices include changing litter input, extending SOC residence time, altering vegetation covers, conducting different tillage practices, etc. In terms of the tillage practice, our model uniformly mixes the top 20 cm in April, before seeding, each year (Table S2). The most significant drawback of tillage is erosion, however, since the gully land topography is quite flat, and check-dam is established to prevent soil erosion, this drawback of tillage is not a predominant concern. Tillage buries the surface-rich SOC into deeper layers, which potentially stores previous carbon-rich soil into a deeper layer with a longer turnover period and provides the capability of newly-exposed surface soils to store more carbon. Therefore, other tillage practices like conventional tillage (i.e.,   5 cm depth) or non-till would not be as effective as the traditional tillage practices (i.e.,   20 cm depth) in this particular landscape. In the gully land consolidation area, different vegetation cover tests are implemented in upland or steep areas where erosion rates are much stronger than the consolidated gully lands^12,37–41^, and the landcover is one of our model inputs (Figure S7a). The different land cover as to protect soil erosion in the gully land is not a major concern in this study, instead, our work aims to test ways to store carbon in soils in consolidated gully lands. Moreover, the majority of the food need in this local region is corn, hence we consider corn as the crop type in our model, and we test different litter input and different SOC residence time on the surface as our study scenarios.

We assess different scenarios related to the biogeochemical decomposition rates in the SCALE model. The possible ways to increase the SOC stock are (1) increasing SOC gain by adding plant residues, and (2) reducing the SOC loss by slowing down the decomposition rate (Eq. 12 in Methods), which corresponds to the input of more stable SOC that has a longer residence time. One approach for the latter is to apply biochar into soils, as many studies have suggested that biochar is capable of increasing SOC content, ameliorating soil fertility decline, and enhancing crop yields^42–45^. A recent study^46^ near Yan’an city in China’s Loess Plateau shows that biochar could increase the SOC content from 4.2 to 9.6 (g C/kg). In our model, we test the impacts of different residence times of surface carbon on total SOC stock change by assigning different decomposition rates in the slow (or humus) pool. We alter decomposition rates in the top 5-cm and let the system evolve in response to this perturbation.

**Figure 6:**
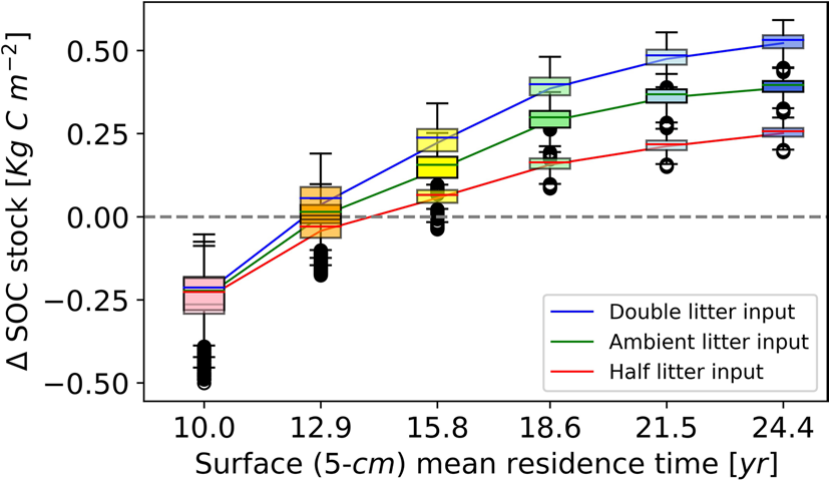
Soil organic carbon stock changes in the consolidated gully associated with different scenarios after a 50-years evolution (see more information in Figure S4 in Supplementary Information). Scenarios include six different mean residence times, at an increment of 2.9 years, from 10 to 24.4 years. Litter input amount includes current, half, and double amount of the current litter input. Each box plot represents the spatial variability of SOC stock change under one management scenario. The current condition is ambient litter input with 12.9 years mean residence time. Each type of litter input is linked using one single solid line. The same mean residence time shares the same color among box plots. Explanation of these box-plots: center line, median; box limits, upper and lower quartiles; whiskers, $1.5\times$ interquartile range.

We test different scenarios through a combination of different mean residence time and different amounts of plant litter input (Figure S5 in Supplementary Information). In general, more litter and longer mean residence time both result in a larger SOC stock increase (Fig. 6). The SOC stock change has a nonlinear relationship with both litter input and residence time in that the SOC approaches towards an asymptotic limit with increases in both. In other words, the increasing rate of SOC stock becomes less sensitive with the increase of litter input or the increase of mean residence time. Moreover, with a longer mean residence time, the SOC stock change is more sensitive to reduced litter input than increased one. Hence, maintaining a current or increased litter input is also critical with more stable biochar applications. In the current land condition, where mean residence time is estimated to be 12.9 years, the SOC content is close to a saturated or upper limit condition based on our simulation. It would result in little increase in the SOC stock by increasing the litter input. Hence, a more effective way to enhance the SOC stock is to apply more stable SOC with a longer mean residence time (i.e., 15.8 years; Fig. 6) with no less than the present litter input. By extending the surface SOC residence time up to 24.4 years, the surface SOC content would have the potential to increase from the current 4.0 $\mathrm{kg\,C}/\mathrm{m}^3$ (Figure S6 in Supplementary Information) to as high as approximately 9.0 $\mathrm{kg\,C}/\mathrm{m}^3$ after a 50-years evolution. After integrating the SOC profile along the vertical soil column, the SOC stock increases 0.5 $\mathrm{kg\,C}/\mathrm{m}^2$, from 2.33 $\mathrm{kg\,C}/\mathrm{m}^2$ (initial) to 2.85 $\mathrm{kg\,C}/\mathrm{m}^2$ (Fig. 6).

## Conclusion

Our analysis shows that the two processes that dictate the SOC dynamics across time and space—transport (or the lateral flux: redistribution of SOC on the surface) and the transformation (or the vertical flux: SOC accumulation of $\hbox {CO}_2$ through plant litterfall and SOC releasing of $\hbox {CO}_2$ via microbial decomposition)—are substantially different between engineered consolidated gullies and the natural watersheds. The SOC transport, which generally follows the pattern of the summer monsoon season, has little impact on the SOC transformation in consolidated gullies but has a strong influence on the SOC transformation in natural watersheds. In natural watersheds, where the spatial mean of SOC stock change is −0.46 $\mathrm{g\,C}/\mathrm{m}^2/\mathrm{year}$, the surface transport is dominant and results in a net erosion (spatial mean: −.85 $\mathrm{g\,C}/\mathrm{m}^2/\mathrm{year}$), and biogeochemical transformation results in a net carbon sink for the atmospheric $\hbox {CO}_2$ (spatial mean: 0.39 $\mathrm{g\,C}/\mathrm{m}^2/\mathrm{year}$). The consolidated gully serves as an enhanced carbon sink in that it not only functions as a net deposition zone of SOC transport ($\sim$ 0.6 $\mathrm{g\,C}/\mathrm{m}^2/\mathrm{year}$) but also a net carbon sink for the atmospheric $\hbox {CO}_2$ ($\sim$ 0.4 $\mathrm{g\,C}/\mathrm{m}^2/\mathrm{year}$).

Since the topography of China’s Loess Plateau has a substantial ravine-like structure, our simulation results of these two watersheds could be linearly upscaled to larger regions. By extending to all of the other consolidated gullies in Yan’an City (approximately 377.8 $\mathrm{km}^2$), the total carbon stock increase rate would be approximately 0.38 $\mathrm{Gg\,C}/\mathrm{year}$ with 0.23 $\mathrm{Gg\,C}/\mathrm{year}$ from SOC deposited from upper land and 0.15 $\mathrm{Gg\,C}/\mathrm{year}$ of the biogeochemical accumulation from atmospheric $\hbox {CO}_2$. Furthermore, we find that extending the surface carbon residence time is more efficient than increasing the litter input into soils in that it has a potential to enhance organic carbon content with a rate as high as 8 $\mathrm{g\,C}/\mathrm{m}^2/\mathrm{year}$ under ambient litter input and 10 $\mathrm{g\,C}/\mathrm{m}^2/\mathrm{year}$ for double the litter input. For the Yan’an city, the initiation region of the gully land consolidation project, careful land management practice would lead to $\hbox {CO}_2$ sequestration of 3.78 $\mathrm{Gg\,C}/\mathrm{year}$ into soils.

In summary, we studied the SOC sustainability in the emergent and massively engineered landscapes under the gully land consolidation project in China’s Loess Plateau. These ‘time zero’ landscapes also present the scientific community to provide an assessment and guidance to ensure sustainability and serve as an exemplar for other such efforts. We used a validated SCALE model to estimate the SOC stock change due to the co-evolution of landscape and biogeochemical transformation over the next 50 years in the consolidated gully and the surrounding natural watersheds. Careful maintenance of the consolidated gullies would result in a sustainable soil condition, which plays a crucial role in ensuring food security, boosting the local economy, and offering more local jobs so that local people would stay instead of pursuing work opportunities in over-populated cities.

## Methods

### SCALE model

Combining the processes of biogeochemical transformation, soil erosion/deposition and resultant landscape evolution, and bioturbation by soil fauna, the governing equations, which conserves SOC mass, can be expressed as^24^:

1 $$\begin{aligned} \frac{ \partial }{ \partial t } \int _{0}^{Z} \mathbf{C } dz =\int _{0}^{Z} \mathbf{g } dz - \nabla \cdot \mathbf{q }_C + \int _{0}^{Z} \nabla \cdot \big [D(z) \nabla \mathbf{C } \big ] dz \end{aligned}$$

where $\mathbf{C }$ is the SOC concentration $[M L^{-3}]$, $\mathbf{C } = [ C_l,C_h,C_b]^T$ represents the fast (or litter), slow (or humus), and microbial biomass pool, respectively; $\mathbf{g }$ is the rate of the biogeochemical transformation process, which is a function of the fast (or litter), slow (or humus), and microbial biomass pool; $\nabla \cdot \mathbf{q }_C$ is the surface SOC flux associated with soil transport; and $D(z)$ is the bioturbation diffusivity. When the vertical column is discretized into several layers, the equations can be written as:

2a $$\begin{aligned} \text {Top soil layer: }&\frac{ \partial \big ( \mathbf{C }_1 z_1 \big ) }{ \partial t } = \mathbf{g }_1 z_1 - \nabla \cdot \mathbf{q }_C \end{aligned}$$

2b $$\begin{aligned} \text {Sublayers: }&\frac{ \partial \mathbf{C }_n }{ \partial t } = \mathbf{g }_n + \nabla \cdot \big [D(z) \nabla \mathbf{C } \big ] dz \end{aligned}$$

where the subscripts 1 and *n* denote the surface soil layer and the $n{\mathrm{^{th}}}$ layer below-surface, respectively. Other details of these equations can be found in^24^.

The mechanisms of soil transport and the resultant landscape evolution can be categorized into two groups: overland flow-driven transport and diffusion-driven transport from other disturbances (e.g., wind, animal, and raindrop splash). The 2-D mass conservation equation of soil transport and the resultant landscape evolution follows Exner equation:

3 $$\begin{aligned} \frac{\partial \eta }{\partial t} = U -\nabla \cdot q_d - \nabla \cdot q_s \end{aligned}$$

where $\eta$ is soil surface elevation [*L*]; *U* is the rate of tectonic uplift or glacial rebound $[L T^{-1}]$; $q_d$ is the volume flux of sediment per unit width by hillslope diffusion $[L^2 T^{-1}]$; $q_s$ is the volume flux of sediment per unit width by overland flow $[L^2 T^{-1}]$.

The diffusion-driven transport $\nabla \cdot q_d$ is a combination of wind erosion, animal disturbance, soil creep, raindrop splash, and biogenic transport. The 2-D equation of $q_d$ is expressed as a linear relationship with slope^47^:

4 $$\begin{aligned} q_d = - D_{x} \frac{ \partial \eta }{\partial x} - D_{y} \frac{ \partial \eta }{\partial y} \end{aligned}$$

where $D_x$ and $D_y$ are the soil diffusion coefficient in *x* and *y* direction, respectively $[L^2 T^{-1}]$. The overland flow-driven transport $\nabla \cdot q_s$ is a combined form of the divergence of stream-power but limited by the detachment capacity^48^:

5 $$\begin{aligned} \nabla \cdot q_s = \min \Bigg ( D_c, \frac{q_{s,out} - \sum q_{s, in}}{d_s} \Bigg ) \end{aligned}$$

where $D_c$ is the detachment capacity, which is the upper limit of of local erosion rate $[L/S]$; $q_{s,out}$ is the sediment flux out of a cell and $\sum q_{s, in}$ is the total sediment flux into a cell assumed at sediment transport capacity.

The rate of change of SOC on the surface driven by soil transport is $\nabla \cdot q_s$, which has a linear relationship with soil transport flux:

6 $$\begin{aligned} \nabla \cdot \mathbf{q }_{C} = \nabla \cdot ( k_{soc} \mathbf{C }_{1} q_d) + \nabla \cdot ( k_{soc} \mathbf{C }_{1} q_s) \end{aligned}$$

where $\mathbf{C } = [C_l , C_h , C_b ]^T$, and the subscript 1 denotes the surface soil layer; $q_d$ and $q_s$ are soil transport flux of diffusion and overland flow; $k_{soc}$ is an enrichment ratio, which represents a preferential transport (mobilization and deposition) of SOC. The SOC fluxes driven by diffusion and overland flow sediment transport are:

7 $$\begin{aligned} \nabla \cdot ( k_{soc} \mathbf{C }_{1} q_d)= & {} - \frac{\partial }{\partial x} \bigg (k_{soc} \mathbf{C }_{1} D_{x} \frac{ \partial \eta }{\partial x} \bigg ) - \frac{\partial }{\partial y} \bigg (k_{soc} \mathbf{C }_{1} D_{y} \frac{ \partial \eta }{\partial y}\bigg ) \end{aligned}$$

8 $$\begin{aligned} \nabla \cdot \big (k_{soc} \mathbf{C }_{1} q_s \big )= & {} \left\{ \begin{array}{ll} k_{soc} \mathbf{C }_{1} D_c , &{}\quad \mathrm{if}\ D_c < \frac{q_{s,out} - \sum q_{s, in}}{d_s} \\ \frac{ k_{soc} \mathbf{C }_{1,out} q_{s,out} - \sum \big ( k_{soc}\mathbf{C }_{1,in} q_{s, in} \big ) }{d_s} , &{}\quad \mathrm{otherwise} \end{array} \right. \end{aligned}$$

The initial conditions include land surface elevation, SOC profiles, soil moisture, and surface water depth for each grid box. Table S1 (in Supplementary Information) lists variables associated with initial conditions. We simulate the SOC surface transport and vertical transformation at a daily time step with 2 $\times$ 2 $\mathrm{m}^2$ spatial resolution on the surface and a range of vertical grid sizes varying from 5 to 60 $\text{cm}$. The initial elevation is from lidar (Light Detection and Ranging) DEM (digital elevation model) with a 2-m resolution, collected by a drone in 2015. The SOC profiles at each 2-D grid are estimated by combining the surface SOC contents from the field survey in 2016 and SOC profiles from soil cores sampled at a nearby site 2-km away (see Section below). We assume that the initial soil thickness is 1-m because the most active soil thickness^49^ regarding SOC and soil moisture are within the top 1 $\text{m}$, and the deeper SOC contents are quite uniform^19,20^, indicating a relatively stable condition. Also, we neglect bedrock weathering, and therefore, the soil thickness change is only from the surface soil erosion or deposition. The SOC stock and soil thickness change in the results are compared with the initial SOC stock in the top 1 m. Other initial values such as soil moisture profile and surface water depth (i.e., initial values assigned spatially uniform at each grid point) have a short-time memory in that the impacts only last for a few days to weeks and are predominantly determined by the external forcing and resulting dynamics (more information in Supplementary Information Table S1).

### Initial soil organic carbon profiles

SOC in a natural setting exponentially decreases with soil depth. Here we assume the following relationship between SOC content and soil depth:

9 $$\begin{aligned} SOC(Z) = a e^{-bZ} + c \end{aligned}$$

where $\text{SOC}(\text{Z})$ represents SOC content at depth $\text{Z}$; $\text{Z}$ is zero at the surface, and positive downward; $a$, $b$, and $c$ are positive and constant parameters, where $a+c$ represents surface SOC content, $b$ represents the decay rate, and $c$ represents the relative stable or immobile SOC at depth.

Parameters $b$ and $c$ in this study are estimated from twenty deep cores—ten sites at hills of the Reference Watershed, eight sites at GLC Watershed in the hills, and two sites in the consolidated gully land (Figure S7a). The SOC contents are shown as dots in Figure S6 (in Supplementary Information). We assume the exponential decay rate ($b$) and the immobile SOC ($c$) are spatially uniform in the cornfield in the consolidated gully area and natural field, respectively. We use the least square non-linear method to fit the sample points, and the fitted curves are solid lines in Figure S6 (in Supplementary Information) with the corresponding relationships obtained as:

10a $$\begin{aligned} \text {For trees/shrubs: }&SOC(Z) = 8.04 e^{-9.63Z}+2.55 \end{aligned}$$

10b $$\begin{aligned} \text {For crops: }&SOC(Z) = 3.71 e^{-7.06Z}+2.27 \end{aligned}$$

hence in the natural area, $b= 9.63$ and $c = 2.55$; and in the cornfield, $b = 7.06$ and $c = 2.27$. The parameter $a$ varies spatially. Soil samples near the soil surface were collected across the whole area of both the GLC and Reference Watershed in 2016. To ensure that the sampling sites are uniformly distributed and represent all land cover types in each watershed, we superimposed an 80-m $\times$ 80-m grid on the DEM map. In each grid cell, we selected one representative site to collect soil samples; in consolidated gullies, adjacent sampling sites were spaced at an interval of  40-m because of the relatively narrow width. SOC was measured in a laboratory by using the dichromate oxidation method^50^. In the GLC Watershed, soil samples were collected at 89 locations with 178 samples (0–10 cm and 10–20 cm); in the Reference Watershed, soil samples were collected at 72 locations with 144 samples (Figure 2a, in Supplementary Information). We used Kriging^51^ to interpolate the SOC content in each 2-D grid box in the two watersheds. Then $a$ is back-calculated with the given values of the two layers at 5 cm and 15 cm (the middle point of the two layers, respectively). The final surface SOC (which equals $a+c$) is shown in Figure S7a (in Supplementary Information).

### Forcing data

To explore the co-evolution of the landscape and the vertical profiles of SOC over the decadal time scale, we target a 50-years simulation. The meteorological data is collected from the China National Field Observation Station in An’sai ($36^{\circ } 51^{\prime } 30^{\prime \prime } N$, $109^{\circ } 19^{\prime } 23^{\prime } E$; data source: http://asa.cern.ac.cn), 44 km away to the northwest from the two watersheds, with a 10 years record from 2008 to 2017, which is the best available data for the simulation. The mean annual precipitation is 560 mm. These data are used to train a stochastic Weather Generator^52^, which is used to create an ensemble of another 40 years of data (Figure S7c in Supplementary Information).

Landcover is also obtained from the field survey in 2015 (Figure S7b in Supplementary Information). It is essential for simulating surface water runoff and the input of SOC from plant residues. Different types of landcover provide different fractions that control the surface water runoff velocity, and such fraction is represented by Manning’s coefficient (Table S2 in Supplementary Information). The plant residues include dead leaves, roots, stems, and corn stover after harvest. Here, we estimate plant residues as a function of the Normalized Difference Vegetation Index (NDVI) (Figure S3a) which is obtained from Landsat satellite data (see more information in the Section below).

### Litter input estimation

The NDVI is collected from Landsat satellite data for a full 2 years period, 2016 and 2017. It is spatially divided into three areas, the consolidated gully land, the GLC Watershed, and the Reference Watershed. The spatial distribution of NDVIs for the two watersheds are nearly identical, so we took the spatial means of NDVI for the two watersheds excluding the consolidated gully to represent the natural area (Figure S3a in Supplementary Information). During the growing season, the NDVIs in a natural area (Figure S3(a1) in Supplementary Information) are smaller than the one in the consolidated gully (Figure S3a2 in Supplementary Information). This is because the crop inside the consolidated gully has higher vegetation density. We assume the rate of litterfall has an exponentially increasing relationship with NDVI (Figure S3b in Supplementary Information). As the NDVI increases, the plant residues increase in general. When a plant’s growth slows down, the NDVI increase also slows down and is close to the maximum, but on the other hand, the litterfall rate increases much faster near the end of the growing season. This characterization allows us to fill in the gap due to the lack of litterfall data about the various land vegetation types, including the cornfield in the consolidated gully.

### Governing equations of SOC transformation

The equation below shows the SOC transformation, which is directly affected by litter input and decomposition rate^29^:

11 $$\begin{aligned} \frac{\partial \big ( C_l + C_h + C_b \big ) }{\partial t} = I_{litter} - \big (r_r K_l C_l+r_r K_h C_h \big ) \end{aligned}$$

where $C_l$, $C_h$, and $C_b$ are defined in Eq. 1; $I_{litter}$ is the litter input from the sum of above-ground litter fall and below-ground root-litter $[M L^{-2}T^{-1} ]$; $r_r$ defines the fraction of decomposed organic carbon to $\hbox {CO}_2$ $[-]$ $(0 \le r_r \le 1-r_h)$, which typically ranges between 0.6 and 0.8; $K_l$ and $K_h$ are rates of carbon decomposition in fast and slow pool, respectively $[T^{-1}]$. They are regulated by soil moisture and carbon-nitrogen ($C/N$) ratio as shown below^29^:

12a $$\begin{aligned}&K_l = \varphi (C/N) f_d(\theta ) k_l C_b \end{aligned}$$

12b $$\begin{aligned}&K_h =\varphi (C/N) f_d(\theta ) k_h C_b \end{aligned}$$

where $k_l$ and $k_h$ represent the rate of decomposition as a simplified term that encompasses different organic components in the litter and humus pool, respectively $[L^3 T^{-1} M^{-1}]$; $\varphi (C/N)$ is a ratio that is from the reduction of the decomposition rate if the immobilization (controlled by nitrogen content) fails to meet the nitrogen demand by the microbes $[-]$. $\varphi \approx 1$ in agricultural fields where nitrogen supply is usually sufficient from fertilizers; $f_d(\theta )$ $[-]$ represents the soil moisture effects on decomposition^29^. The optimal soil moisture condition is the field capacity which provides the highest $f_d$^29^, meaning that very dry or very wet conditions will result in a smaller $f_d$, and hence reduce the decomposition rate. The decomposition rates for litter (or fast) pool and humus (or slow) pool from the equations are $r_r K_l$ and $r_r K_h$, respectively. In this study, we test the different mean residence times on the surface soil layer (5-cm) by assigning a new decay rate of the decomposition parameter $k_h$ in humus (or slow) pool. A complete list of parameters can be found in Supplementary Information Table S2.

## Supplementary information

Supplementary Information.

## Data Availability

The Landsat 7 ETM+ C1 Level-1 data product was retrieved from the online Data Pool, courtesy of the NASA Land Processes Distributed Active Archive Center (LP DAAC), USGS/Earth Resources Observation and Science (EROS) Center, Sioux Falls, South Dakota, https://earthexplorer.usgs.gov/. The 10-years recorded meteorological data was retrieved from China National Field Observation Station in An‘sai, http://asa.cern.ac.cn. LiDAR DEM cannot be accessed directly due to the China government policy but can be acquired through Loess and Quaternary Geology, Institute of Earth Environment, Chinese Academy of Sciences, http://paleodata.ieecas.cn. Landcover, spatial surface SOC content from field survey, and soil core samples used in this research are openly available at https://github.com/HydroComplexity/SCALE-GLC.

## Code availability

The SCALE model is openly available at https://github.com/HydroComplexity/SCALE.

## References

[1] Shi H, Shao M (2000). Soil and water loss from the Loess Plateau in China. J. Arid Environ..

[2] Liu Q, Wang Y, Zhang J, Chen Y (2013). Filling gullies to create farmland on the Loess Plateau. Environ. Sci. Technol..

[3] Zhao G (2016). Evidence and causes of spatiotemporal changes in runoff and sediment yield on the Chinese Loess Plateau. Land Degrad. Dev..

[4] He C (2015). The situation, characteristics and effect of the gully reclamation project in Yanan. J. Earth Environ..

[5] Liu Y, Li Y (2017). Engineering philosophy and design scheme of gully land consolidation in Loess Plateau. Trans. Chin. Soc. Agric. Eng..

[6] Jin Z (2019). Valley reshaping and damming induce water table rise and soil salinization on the Chinese Loess Plateau. Geoderma.

[7] HPRC. Grain for green project (2009). http://www.hprc.org.cn/gsgl/dsnb/zdsj/200912/t20091230_39594.html.

[8] Chen Y (2015). Balancing green and grain trade. Nat. Geosci..

[9] Li Y (2016). Reduced runoff due to anthropogenic intervention in the Loess Plateau, China. Water (Switzerland).

[10] Li S (2016). Vegetation changes in recent large-scale ecological restoration projects and subsequent impact on water resources in Chinas Loess Plateau. Sci. Total Environ..

[11] Liang W (2015). Quantifying the impacts of climate change and ecological restoration on streamflow changes based on a Budyko hydrological model in Chinas Loess Plateau. Water Resour. Res..

[12] Feng X, Fu B, Lu N, Zeng Y, Wu B (2013). How ecological restoration alters ecosystem services: an analysis of carbon sequestration in Chinas Loess Plateau. Sci. Rep..

[13] Feng X, Cheng W, Fu B, Lu Y (2016). The role of climatic and anthropogenic stresses on long-term runoff reduction from the Loess Plateau, China. Sci. Total Environ..

[14] Wilson CG (2018). The intensively managed landscape critical zone observatory: a scientific testbed for understanding critical zone processes in agroecosystems. Vadose Zone J..

[15] Kimble, J. M. *et al.**Soil Carbon Management: Economic, Environmental and Societal Benefits* (Faculty & Staff Authored Books, Boca Raton, 2007).

[16] Lal R, Follett RF (2009). Soil Carbon Sequestration and the Greenhouse Effect.

[17] Milne E (2015). Soil carbon, multiple benefits. Environ. Dev..

[18] Feng X (2016). Revegetation in Chinas Loess Plateau is approaching sustainable water resource limits. Nat. Clim. Change.

[19] Zhao Y (2019). Exploring the role of land restoration in the spatial patterns of deep soil water at watershed scales. Catena.

[20] Wang Y, Han X, Jin Z, Zhang C, Fang L (2016). Soil organic carbon stocks in deep soils at a watershed scale on the Chinese Loess Plateau. Soil Sci. Soc. Am. J..

[21] Vanwalleghem T, Stockmann U, Minasny B, Mcbratney AB (2013). A quantitative model for integrating landscape evolution and soil formation. J. Geophys. Res. Earth Surf..

[22] Temme AJ, Vanwalleghem T (2016). LORICA: a new model for linking landscape and soil profile evolution—development and sensitivity analysis. Comput. Geosci..

[23] Finke PA, Hutson JL (2008). Modelling soil genesis in calcareous loess. Geoderma.

[24] Yan Q (2019). Three-dimensional modeling of the coevolution of landscape and soil organic carbon. Water Resour. Res..

[25] Yue Y (2016). Lateral transport of soil carbon and land-atmosphere CO$$_2$$ flux induced by water erosion in China. Proc. Natl. Acad. Sci..

[26] Zhang H (2014). Inclusion of soil carbon lateral movement alters terrestrial carbon budget in China. Sci. Rep..

[27] Yan Q (2017). Hydrogeomorphological differentiation between floodplains and terraces. Earth Surf. Process. Landf..

[28] Wang T (2010). Ecologically asynchronous agricultural practice erodes sustainability of the Loess Plateau of China. Ecol. Appl..

[29] Porporato A, DOdorico P, Laio F, Rodriguez-Iturbe I (2003). Hydrologic controls on soil carbon and nitrogen cycles. I. Modeling scheme. Adv. Water Resour..

[30] Van Oost K (2007). The impact of agricultural soil erosion on the global carbon cycle. Science.

[31] Berhe AA, Harden JW, Torn MS, Harte J (2008). Linking soil organic matter dynamics and erosion-induced terrestrial carbon sequestration at different landform positions. J. Geophys. Res. Biogeosci..

[32] Berhe AA (2012). Persistence of soil organic matter in eroding versus depositional landform positions. J. Geophys. Res. Biogeosci..

[33] Dialynas YG (2016). Topographic variability and the influence of soil erosion on the carbon cycle. Glob. Biogeochem. Cycles.

[34] Billings SA, Richter DB, Ziegler SE, Prestegaard K, Wade AM (2019). Distinct contributions of eroding and depositional profiles to land-atmosphere CO$$_2$$ exchange in two contrasting forests. Front. Earth Sci..

[35] An Z, Kukla GJ, Porter SC, Xiao J (1991). Magnetic susceptibility evidence of monsoon variation on the Loess Plateau of central China during the last 130,000 years. Quat. Res..

[36] Doetterl S (2016). Erosion, deposition and soil carbon: a review of process-level controls, experimental tools and models to address C cycling in dynamic landscapes. Earth Sci. Rev..

[37] Fu B (1989). Soil erosion and its control in the Loess Plateau of China. Soil Use Manag..

[38] Chen L (2007). Effect of land use conversion on soil organic carbon sequestration in the loess hilly area, Loess Plateau of China. Ecol. Res..

[39] Fu B, Chen L, Ma K, Zhou H, Wang J (2000). The relationships between land use and soil conditions in the hilly area of the loess plateau in northern Shaanxi, China. Catena.

[40] Fang X, Xue Z, Li B, An S (2012). Soil organic carbon distribution in relation to land use and its storage in a small watershed of the Loess Plateau, China. Catena.

[41] Zhao C, Jia X, Zhu Y, Shao M (2017). Long-term temporal variations of soil water content under different vegetation types in the Loess Plateau, China. Catena.

[42] Novak JM (2009). Impact of biochar amendment on fertility of a southeastern coastal plain soil. Soil Sci..

[43] Major J, Rondon M, Molina D, Riha SJ, Lehmann J (2010). Maize yield and nutrition during 4 years after biochar application to a Colombian savanna oxisol. Plant Soil.

[44] Atkinson CJ, Fitzgerald JD, Hipps NA (2010). Potential mechanisms for achieving agricultural benefits from biochar application to temperate soils: a review. Plant Soil.

[45] Jeffery S, Verheijen FG, van der Velde M, Bastos AC (2011). A quantitative review of the effects of biochar application to soils on crop productivity using meta-analysis. Agric. Ecosyst. Environ..

[46] Su C-C, Ma J-F, Chen Y-P (2019). Biochar can improve the soil quality of new creation farmland on the Loess Plateau. Environ. Sci. Pollut. Res..

[47] Furbish DJ, Fagherazzi S (2001). Stability of creeping soil and implications for hillslope evolution. Water Resour. Res..

[48] Yetemen O, Istanbulluoglu E, Flores-Cervantes JH, Vivoni ER, Bras RL (2015). Ecohydrologic role of solar radiation on landscape evolution. Water Resour. Res..

[49] Wang Y (2015). Soil organic carbon in deep profiles under Chinese continental monsoon climate and its relations with land uses. Ecol. Eng..

[50] Nelson D, Sommer L (1982). Total Carbon, Organic Carbon and Organic Matter. Methods of Soil Analysis, Part 2: Chemical and Microbiological Properties.

[51] Gotway CA, Ferguson RB, Hergert GW, Peterson TA (1996). Comparison of Kriging and inverse-distance methods for mapping soil parameters. Soil Sci. Soc. Am. J..

[52] Ivanov, V. Y., Bras, R. L. & Curtis, D. C. A weather generator for hydrological, ecological, and agricultural applications. *Water Resour. Res.* **43**, W10406. 10.1029/2006WR005364 (2007).

