# Supplementary material for: Sustainability of soil organic carbon in consolidated gully land in China’s Loess Plateau
Source: Sci Rep. 2020 Oct 9;10:16927. doi: 10.1038/s41598-020-73910-7 (PMC7547692; doi:10.1038/s41598-020-73910-7)
Supplement: Supplementary file 1 — Supplementary Information. [file 41598_2020_73910_MOESM1_ESM.pdf]

Supplementary Information: Sustainability of Soil Organic Carbon in Consolidated Gully Land in China's Loess Plateau

Qina Yan[1,2], Praveen Kumar*[1,3], Yunqiang Wang[4,5], Yali Zhao[4,5], Henry Lin[6,7], Qihua Ran[8], Zhisheng An[4,5], & Weijian Zhou[4,5]

[1]*Department of Civil and Environmental Engineering, University of Illinois at Urbana-Champaign, Urbana, Illinois, USA.*
[2]*Now at Lawrence Berkeley National Laboratory, Berkeley, California, USA*
[3]*Department of Atmospheric Sciences, University of Illinois at Urbana-Champaign, Urbana, Illinois, USA.*
[4]*State Key Laboratory of Loess and Quaternary Geology, Institute of Earth Environment, Chinese Academy of Sciences, Xi'an, Shaanxi, China*
[5]*CAS Center for Excellence in Quaternary Science and Global Change, Chinese Academy of Sciences, Xi'an, China.*
[6]*Department of Ecosystem Science and Management, Pennsylvania State University, University Park, Pennsylvania, USA.*
[7]*Deceased*
[8]*Institute of Hydrology and Water Resources, College of Civil Engineering and Architecture, Zhejiang University, Hangzhou, China.*

Soil and SOC transport within watersheds and the consolidated gullies

Figure S1 shows soil thickness changes after 50 years evolution in the Reference watershed, GLC (gully land consolidation) watershed excluding the consolidated gully, and the consolidated gully, respectively. Here we assume a zero weathering rate for the soil column in the loess plateau region[1], and, therefore, the thickness change is from the surface soil erosion or deposition only. Severe erosion happens at the sharp slope transition in the upland gullies for both watersheds. However, the soils detached from erosional sites are mostly deposited at a nearby location within the watersheds possibly due to micro-depressions. Hence, the soil thickness change has a relatively large spatial range compared to the spatial mean values. In general, the consolidated gullies is under a depositional environment. The strongest deposition zone is near the two edges of consolidated gullies along the upland. There is no clear strong erosion zone in the consolidated gullies. Moderate erosion and deposition happen throughout the consolidated gullies.

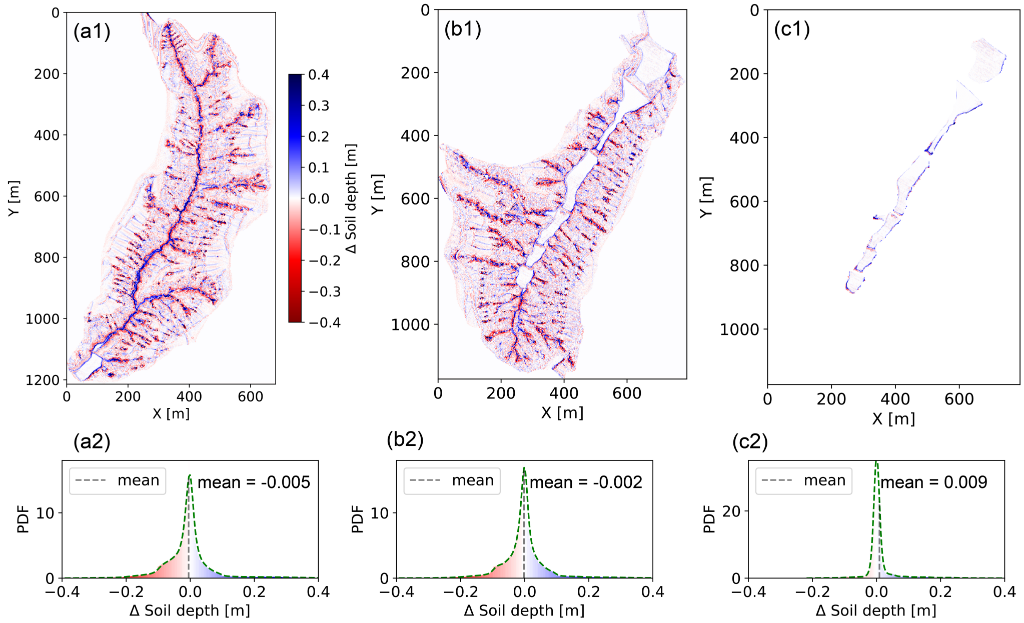

Figure S1: The total soil depth change after 50 years of co-evolution in the (a1) Reference Watershed, (b1) GLC Watershed, and (c1) consolidated gully land. The corresponding probability distribution functions for the spatial variability of Reference Watershed, GLC Watershed, and consolidated gully are shown in (a2), (b2), and (c2), respectively.

Comparison of Soil Organic Carbon transport and transformation

The SOC stock change at each of the 2×2 m^2 grid box is the net of the surface SOC transport and SOC biogeochemical transformation. The surface SOC transport is due to lost through erosion or gained through deposition, hence referred as the lateral flux. The SOC biogeochemical transformation is due to the soil-atmosphere CO_2 exchange that includes indirect accumulation via plant residues or release to the atmosphere as CO_2 through microbial decomposition [2–4], hence referred as the vertical flux. Figure S2a shows the stocks and flows of carbon through different reservoirs, which are air, plants, and soils. Figure S2b (expanded from Figure 4a in the main text) uses the data from Reference Watershed to show the lateral transport (x-axis) and vertical transformation (y-axis) relationship at each 2-D grid box. Within this domain, it can be divided into six groups, and each group has different legacies of SOC stock change:

1. $x > 0$, $y > 0$: ultimate sink (blue). SOC transport results in a net deposition and biogeochemical transformation results in a net carbon accumulation.
2. $x > 0$, $y < 0$, and $|x| > |y|$: deposition dominated (green); SOC accumulation $<$ decomposition; the site is a source to the atmospheric CO_2.
3. $x < 0$, $y > 0$, $|y| > |x|$: transformation dominated (aqua); SOC accumulation $>$ decomposition; the site is a sink to the atmospheric CO_2.
4. $x < 0$, $y < 0$: ultimate source (red). SOC transport results in a net erosion and biogeochemical transformation results in a net carbon loss.
5. $x < 0$, $y > 0$, $|x| > |y|$: erosion dominated (orange); SOC accumulation $>$ decomposition; the site is a sink to the atmospheric CO_2.
6. $x > 0$, $y < 0$, $|y| > |x|$: transformation dominated (yellow); SOC accumulation $<$ decomposition; the site is a source to the atmospheric CO_2.

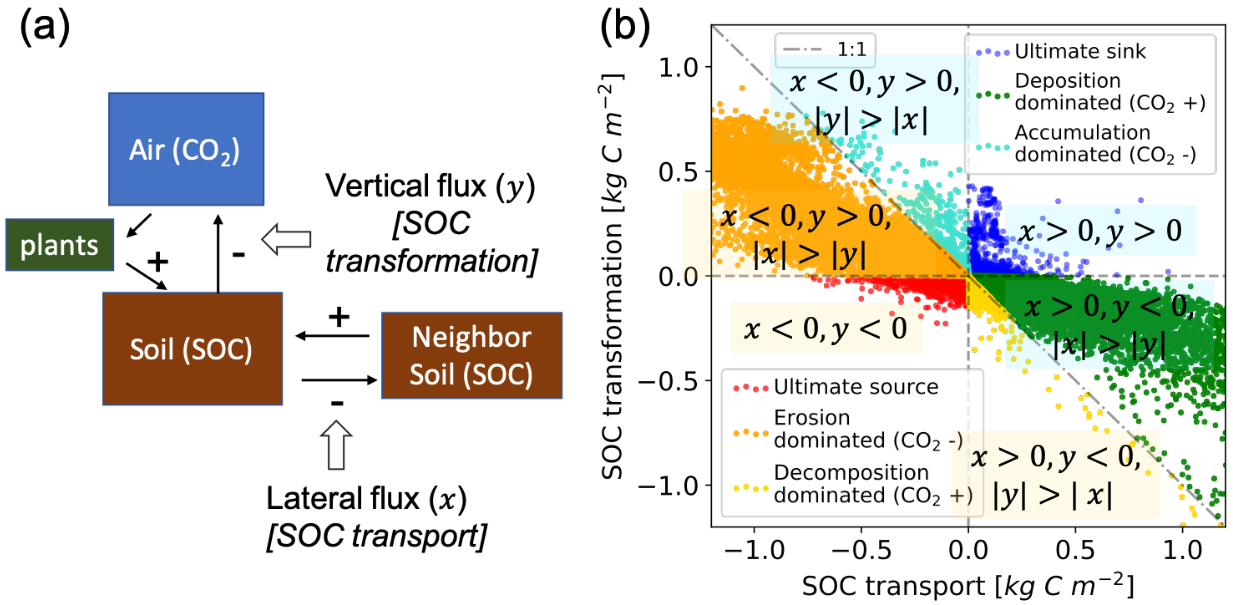

Figure S2: Illustration of the contribution of Soil Organic Carbon (SOC) transport and transformation at each grid point. a) Conceptual illustration of stocks and flows of carbon between atmosphere, soil, and plants. b) Illustration of six groups with different signs and relative magnitudes in the SOC transport and transformation dynamics.

Litter input estimation

The litter input is estimated from the Normalized Difference Vegetation Index (NDVI) (Figure S3a). The relationship between surface litter input and NDVI follows an exponential relationship.

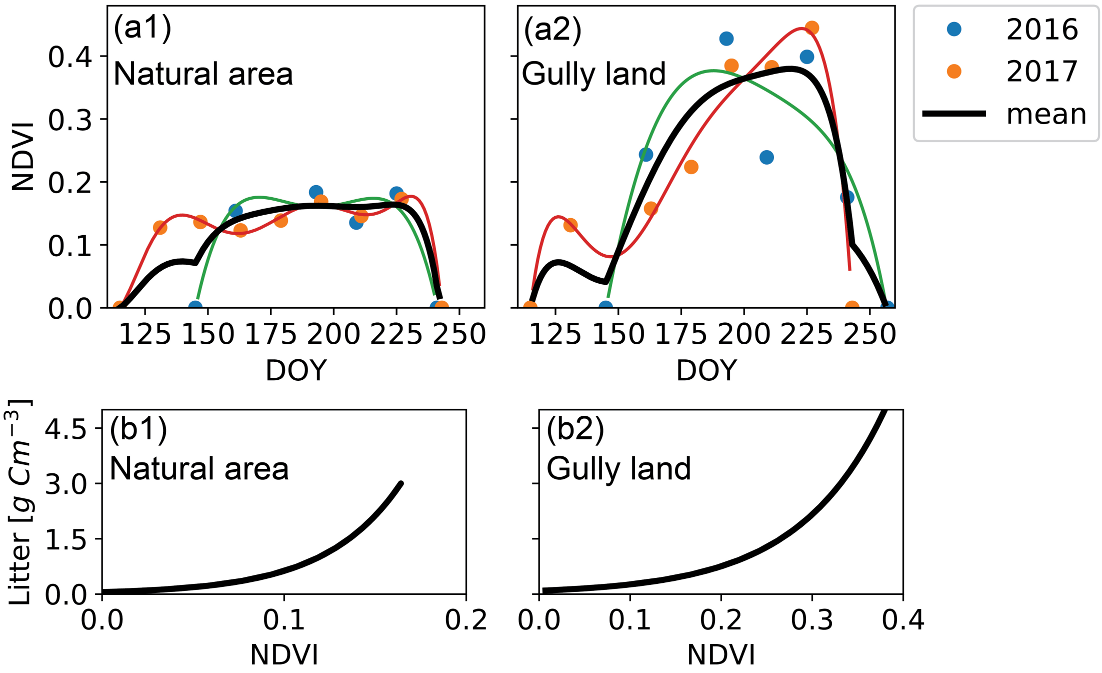

Figure S3: Normalized difference vegetation index (NDVI) and associated litter input. a) NDVI is processed from Landsat satellite bands for two years of record (2016-2017). The plot shows spatial average for the natural (or watersheds) area is spatially averaged for the natural area (a1) and consolidated gully land (a2), respectively. The black line is the mean for the 2 years record, which is then used through the simulation period repeatedly for each year. b) The estimated relationship between the above ground input of plant residues and NDVI.

Depositional zone and erosion zone

Within the PDF of the Reference watershed (Figure S1(a2)), two zones (erosional and depositional zone) are chosen as shown in Figure S4. The spatial means of the fluxes of SOC transport and transformation are extracted to reveal the SOC intra-annual cycle under soil erosion and deposition conditions. The results are shown in Figure 5(b2&b3) in the main text.

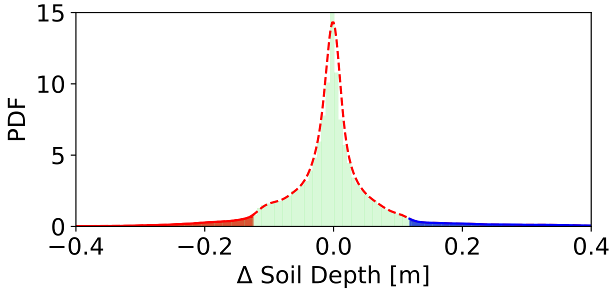

Figure S4: Two zones from the probability density function of total soil depth changes. Each zone accounts for 5% of the total watershed area.

Land management practices in the consolidated gullies

Comparative study of set different scenarios which have different combinations of litter input and SOC mean residence time in the surface ($5\ cm$) soil to test how SOC stock changes after a 50-yr evolution (Figure S5).

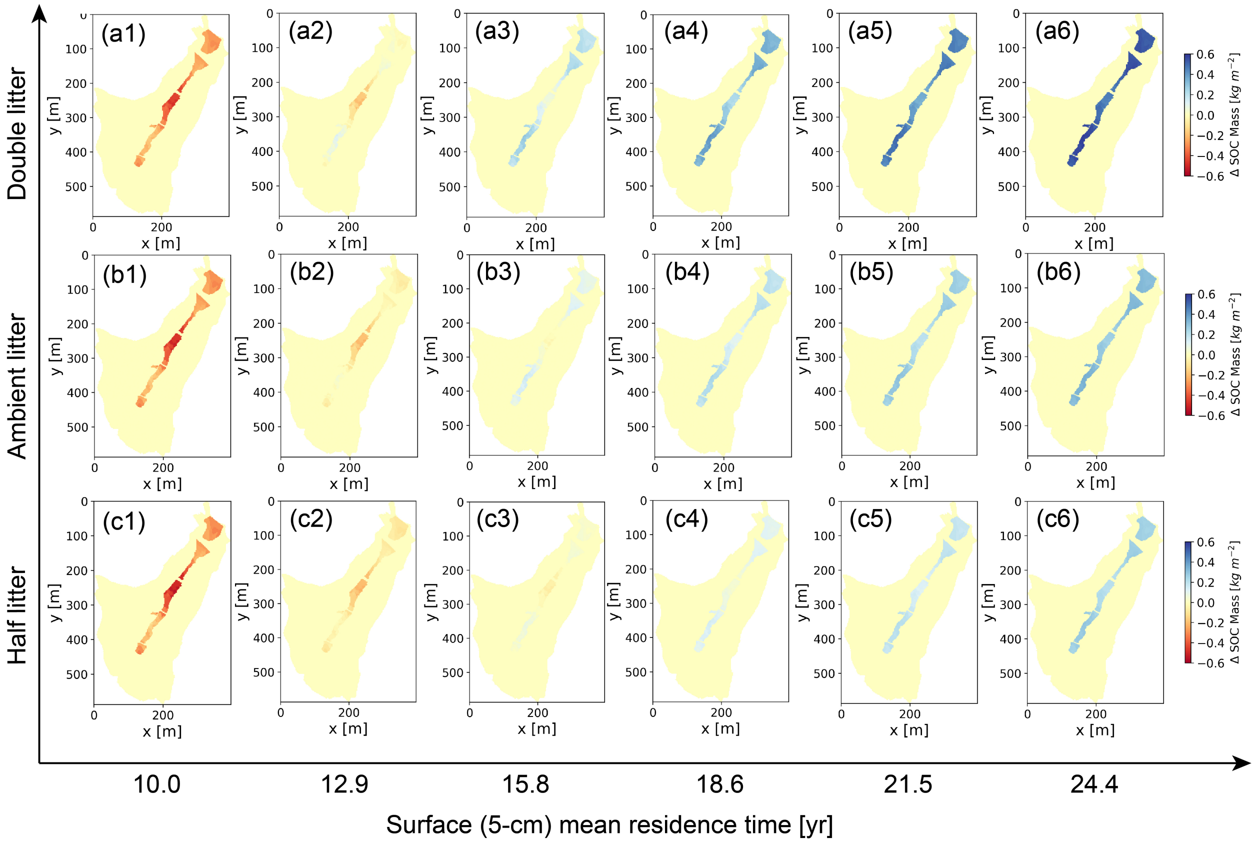

Figure S5: SOC stock changes in the consolidated gully land due to different land management practices. (a) Double the litter input into the consolidated gullies. (b) The current litter input. (c) Litter input reduced by half of the current value. (1)-(6) Application of biochar to soils with different mean residence times. The current condition is (b2).

Initial Soil Organic Carbon profiles

Figure S6 shows the estimation of the initial SOC profile in the Gutun study sites. We collected twenty profiles which includes SOC content and bulk density. Among the twenty sites, eighteen sites are with natural plants and two sites are with corn field. Details of the empirical relationship between SOC concentration and soil depth can be found in the Methods Section.

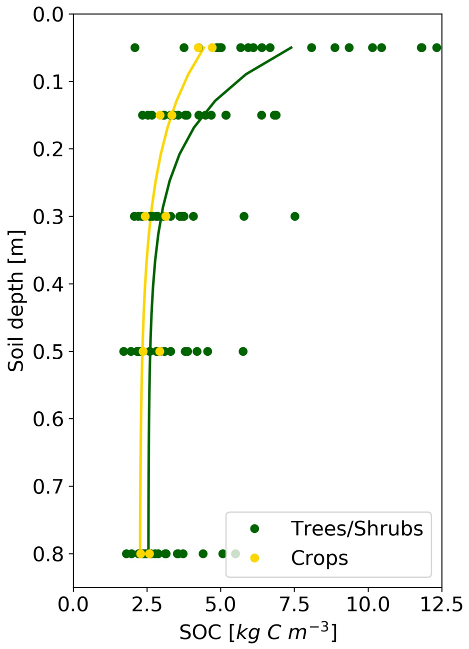

Figure S6: Illustration of the estimation of initial SOC profiles. Dots are from field sampling as shown in Figure S7a. Solid lines are fitted curve using the non-linear least square method for $SOC(Z) = ae^{-bZ} + c$, where a, b, and c are parameters, and Z is the soil depth measured from the surface.

Surface SOC and forcing data

The spatial map of surface SOC and land cover are from the field survey in 2015. SOC is sampled at 0-10 cm and 10-20 cm at the locations as shown in Figure S7a. The surface values of SOC is based on the SOC profiles (Figure S6) when the soil thickness is zero. The rainfall data with 10-yr record and 40-yr simulation are shown in the Figure S7c.

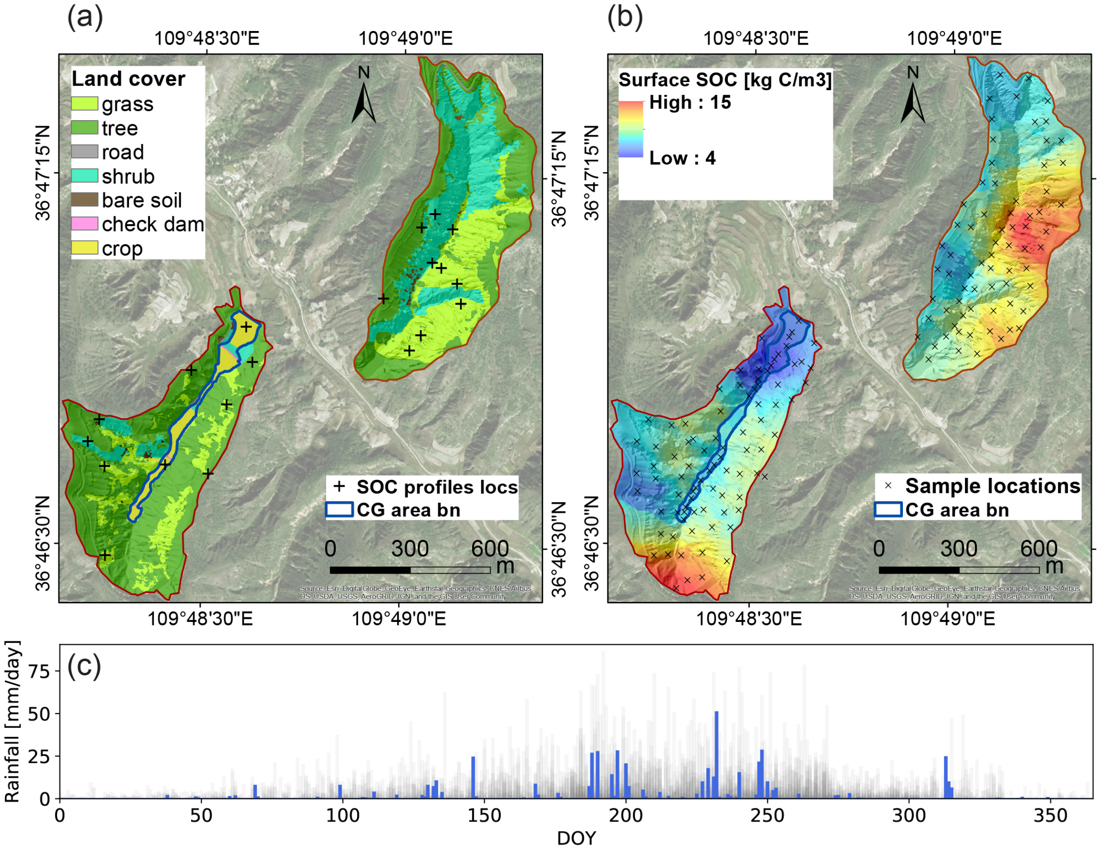

Figure S7: a) Surface soil organic carbon (SOC) concentration from the field survey conducted in 2015. The survey locations are identified as points on the map. The surface SOC is the initial SOC for the SCALE model [5]. b) Vegetation cover is also obtained from the field survey in 2015. This vegetation cover determines the roughness coefficient for surface overland flow and plant residue input into soils. Created using ArcGIS version 10.5. c) Daily precipitation for ten years of observation of record (2008-2017) and an additional 40 years of the simulation are shown as gray bars. The highlighted bars in blue illustrate the observed precipitation in the year 2017 as an illustration of intra-annual variability. The rainfall data are collected by China National Field Observation Station in An'sai city ($36^o51`30''N$, $109^o19`23''E$), 44 km away on the northwest side of our study site (Gutun station), which is the closest available station.

Variables and initial values to the SCALE model

Table S1: Variables and initial values used in the case study

Variables	Symbol	Units	Initial value
Sediment transport variables			
Land surface elevation	η	m	DEM (Digital Elevation Model)
Soil depth of each layer	Z	m	0.05, 0.11, 0.19, 0.29, 0.42, 0.62, 1.0[a]
Overland flow variables			
Surface water elevation	H	m	same as DEM
Surface water depth	h	m	0.0
Soil moisture variables			
pressure head	Ψ	m	-2.7 at each layer
soil moisture	θ	[–]	0.46 at each layer
Soil organic matter parameters			
Carbon in fast (or litter) pool	C_l	$kg\ C/m^3$	see footnote[b]
Carbon in slow (or humus) pool	C_h	$kg\ C/m^3$	see footnote[b]
Carbon in biomass pool	C_b	$kg\ C/m^3$	see footnote[b]

[a]from surface to bottom of the initial 1 m soil depth for each layer. The distance among each other is the thickness for each layer.
[b] The carbon concentration at each grid is a function of soil depth(Z) following the profile as shown in Figure S6.

Input parameters to the SCALE model

Table S2: Parameters of SCALE model

Parameter	Symbol	Units	Value
Overland Flow			
Manning's value for grass, tree, road, shrub, bare soil, check-dam, and crop, respectively	n	$s/m^{1/3}$	0.094, 0.125, 0.034, 0.188, 0.078, 0.282, 0.125[a]
Soil Moisture			
saturated water content, or porosity	θ_s	[–]	0.477[a]
saturated hydraulic conductivity	K_{sat}	m/day	4.8×10^{-4a}
residual water content	θ_r	[$1/m$]	0.08
specific storage coefficient	S_s	[–]	5×10^{-4}
field capacity	θ_{fc}	[–]	0.143
soil surface evaporation rate	E_s	m/day	$3.2 \times 10{-}4$
plant total transpiration rate	T_{max}	m/day	9.1×10^{-4}
Soil Organic Matter			
litter at harvest on surface (corn)	$I_{litter}^{sf,h}$	$kg\,C/m^3$	450[b]
litter at harvest below surface (corn)	$I_{litter}^{bg,h}$	$kg\,C/m^3$	200[b]
decomposition coefficient for fast (or litter) pool	k_l	$m^3/day/g\,C$	see footnote[c]
decomposition coefficient for slow (or humus) pool	k_h	$m^3/day/g\,C$	see footnote[c]
death rate of microbial biomass	k_{rd}	$1/day$	see footnote[c]
bioturbation diffusivity on surface	D_{top}	m^2/yr	$(4 \times 10^{-4})^d$
Sediment Transport			
soil linear diffusion coefficient	D_x, D_y	m^2/yr	0.024 and 0.024
critical shear stress	τ_c	$km/m/s^2$	5.6[e]
rill erosion coefficient	K_r	s/m	0.005[e]
sheet erosion coefficient	K_{qs}	[–]	0.00015[e]
rill erosion coefficient	α	[–]	1.6[e]
soil bulk density	ρ_s	kg/m^3	1.34×10^{3e}
uplift rate	U	m/yr	0.0
soil weathering rate	P	m/yr	0.0
Tillage			
plowing depth	Z_m	m	0.20
tillage time each year	DOY_{till}	day	105

[a] field survey in 2015

[b] 5

[c] k_l, k_h, and k_{rd} are solved by assuming a steady state condition of SOC.

[d] 6

[e] 7

References

1. Wang, Y., Han, X., Jin, Z., Zhang, C. & Fang, L. Soil organic carbon stocks in deep soils at a watershed scale on the Chinese Loess Plateau. *Soil Science Society of America Journal* **80**, 157–167 (2016).

2. Porporato, A., D'Odorico, P., Laio, F. & Rodriguez-Iturbe, I. Hydrologic controls on soil carbon and nitrogen cycles. I. Modeling scheme. *Advances in Water Resources* **26**, 45–58 (2003).

3. Van Oost, K. *et al.* The impact of agricultural soil erosion on the global carbon cycle. *Science* **318**, 626–629 (2007).

4. Berhe, A. A., Harden, J. W., Torn, M. S. & Harte, J. Linking soil organic matter dynamics and erosion-induced terrestrial carbon sequestration at different landform positions. *Journal of Geophysical Research: Biogeosciences* **113**, 1–12 (2008).

5. Yan, Q. *et al.* Three-Dimensional Modeling of the Coevolution of Landscape and Soil Organic Carbon. *Water Resources Research* **55**, 1218–1241 (2019).

6. Quijano, J. C., Kumar, P. & Drewry, D. T. Passive regulation of soil biogeochemical cycling by root water transport. *Water Resources Research* **49**, 3729–3746 (2013).

7. Ma, H. *et al.* The exceptional sediment load of fine-grained dispersal systems: Example of the Yellow River, China. *Science Advances* **3**, 1–8 (2017).
